# Electric fields generated by synchronized oscillations of microtubules, centrosomes and chromosomes regulate the dynamics of mitosis and meiosis

**DOI:** 10.1186/1742-4682-9-26

**Published:** 2012-07-02

**Authors:** Yue Zhao, Qimin Zhan

**Affiliations:** 1 State key laboratory of molecular oncology, Cancer Institute & Hospital of Chinese Academy of Medical Sciences, Peking Union Medical College, Room 6107, No.17 Pan Jia Yuan Nan Li, Chao Yang District, Bei Jing, 100021, China

**Keywords:** Polar wind, Chromosome, Microtubule, Centrosome, Spindle body

## Abstract

Super-macromolecular complexes play many important roles in eukaryotic cells. Classical structural biological studies focus on their complicated molecular structures, physical interactions and biochemical modifications. Recent advances concerning intracellular electric fields generated by cell organelles and super-macromolecular complexes shed new light on the mechanisms that govern the dynamics of mitosis and meiosis. In this review we synthesize this knowledge to provide an integrated theoretical model of these cellular events. We suggest that the electric fields generated by synchronized oscillation of microtubules, centrosomes, and chromatin fibers facilitate several events during mitosis and meiosis, including centrosome trafficking, chromosome congression in mitosis and synapsis between homologous chromosomes in meiosis. These intracellular electric fields are generated under energy excitation through the synchronized electric oscillations of the dipolar structures of microtubules, centrosomes and chromosomes, three of the super-macromolecular complexes within an animal cell.

## Background

The choreography of microtubules, centrosomes and chromosomes during mitosis and meiosis is beautifully designed by nature. Finely regulated and synchronized movements of these super-macromolecular complexes against the entropic forces within a dividing cell ensure the fidelity of the genetic material in both daughter cells. Currently, several models exist for the mechanisms of chromosome congression and spindle body assembly during M phase such as the search and capture model, kinetochore-mediated k-fibre formation, kinetochore motors contributing to congression, and the polar wind model. The mechanisms evoked by these models probably overlap, so there is redundancy among them, since mutations in the genes involved have only mild effects on chromosome congression during mitosis [1]. Many open questions remain within these models. In the polar-wind model, an unknown force (also known as the ejection force) generated by the spindle poles is considered to push the chromosomes to the spindle equator. Laser microsurgery experiments show that chromosome fragments without kinetochores are invariably expelled from the spindle, and chromosomes without kinetochores can still move from the vicinity of the spindle pole to the spindle equator [1-3]. The ejection force of the spindle body is dependent on the polymerization of spindle body microtubules, as depolymerization of astral microtubules by nocodazole or colcemid prevents the expulsion of severed chromosome arms from the spindle, whereas stabilization of microtubules by taxol drives chromosomes to the periphery of the astral array [4]. In addition, the driving force responsible for the pole-ward flux of spindle microtubules during metaphase remains uncharacterized [5].

Cellular electric fields have been studied in various cell types, and several studies have reported the existence of dielectrophoretic forces around cells [6-8]; electromagnetic interactions between cells have also been studied [9-11]. Cifra et al. proposed that microtubules, which comprise heterodimers polymerized into a helical structure, can generate an electric field under intracellular energy excitation [12-15]. Inhibition of microtubule polymerization by an external electromagnetic field has been reported by Kirson et al. [16,17]. Pokorny´ et al. detected four peaks of electric field activity around yeast cells during M phase, which correlated with spindle body assembly, kinetochore microtubule capture, and mitotic spindle elongation during anaphase A and B, visualized by fluorescence microscopy [18]. Comparing synchronized and unsynchronized tubulin mutants of yeast cells, Pokorny´ et al. verified that synchronized yeast cells show more electric activity during M phase than non-synchronized yeasts [19]. Direct measurements of electric resonant oscillations in microtubules have been presented at conferences by A. Bandyopadhyay. The technical aspects of direct detection of electric fields within a living cell have been discussed in a recent review [20]. Resonance absorption of external electromagnetic fields by cancers has been reported by Vedruccio et al. [21], and Zimmerman et al. reported that cancer cell proliferation is inhibited by specific modulation frequencies [22].

Coherent oscillations in microtubules can be explained by Fröhlich’s theory, which describes a system of oscillators with energy supply, linear and nonlinear coupling with a heat bath. If a sufficient energy supply is provided to this system, condensation of energy occurs in the lowest mode leading to its coherent excitation [23,24]. Electrostatic but not electrodynamic interactions are screened over long distances (Debye Screening). Given an intracellular salt concentration of ~ 150 mM, the effectiveness of electrostatic interaction is shortened to the nanometer range (the Debye length is ~ 0.7-0.8 nm). However, resonant electrodynamic interactions, such as the electromagnetic interactions generated by electric oscillations within the cell, may play a role in the long-distance recruitment of biomolecules. Following Fröhlich, Preto et al. suggested that long-range electrodynamic interactions can be triggered only under resonance conditions, and such interactions are effective when one normal mode is statistically privileged, typically out of thermal equilibrium, which could be the case in the intracellular context [25,26].

In this article, we integrate research from several disciplines to provide an ‘electric’ view of the dynamics of these super-macromolecular complexes in mitosis, meiosis and other relevant cellular events. From our theoretical point of view, many of the unidentified forces regulating major cellular dynamic events during mitosis are probably electric forces generated by the synchronized oscillation of the electric dipoles within these super-macro organelle structures. Chronic exposure to extremely low frequency electric fields could affect several key steps of mitosis and neuronal cell physiology, resulting in an increased risk for cancer.

### The electrical properties of microtubules and centrosomes

**Figure 1:**
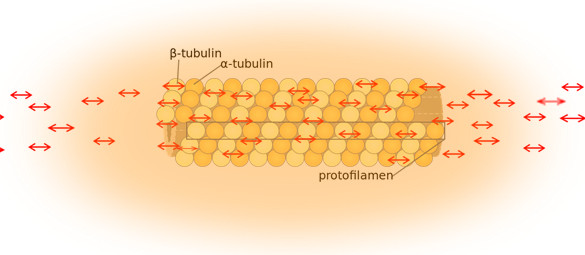
**The red arrow illustrates the electric field of the microtubule under intracellular energy excitation** .

The electric field of the microtubule is generated by the synchronized oscillation of α and β tubulins. These tubulins form electric dipoles during microtubule polymerization; under intracellular energy excitation, synchronized oscillation of α and β tubulin subunits generates a longitudinal electric field around the microtubule [12-15] (Figure 1). Cifra et al. suggested that the source of the energy excitation could be hydrolysis of guanosine triphosphate (GTP) during the process of dynamic instability of microtubules, and also energy transferred from the movement of motor proteins or released from mitochondria as “wasted” energy from the citric acid cycle. We propose that the overall entropic environment within a living cell could be the source of energy for electric oscillation of microtubules. Cancer cells have different entropic states from normal cells as a result of the Warburg effect, which can cause mitochondrial malfunctions and further lead to alteration of cytoskeleton-based cellular elastoelectrical oscillations [27]. The microtubule networks of cancer cells generate an electromagnetic field with different frequencies. Thus, specific electromagnetic frequencies have been used to diagnose specific cancers [21,28], and tumor-specific modulating electromagnetic fields have been used to treat patients with advanced cancer with positive results [22,29]. 

**Figure 2:**
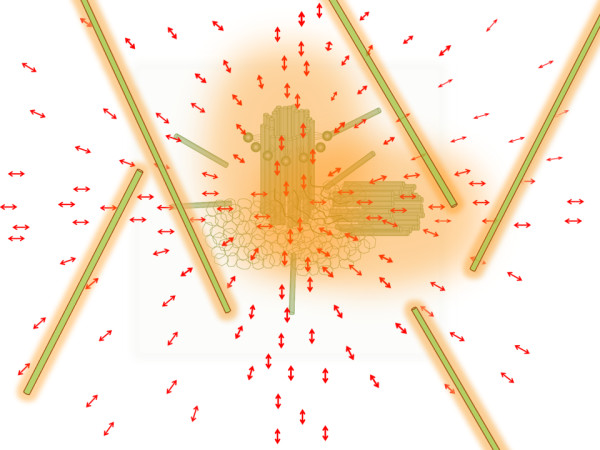
**The red arrows illustrate the electric field of a centrosome under intracellular energy excitation** .

The centriole of the centrosome is composed of α, β and γ tubulins organized differently from the subunits of microtubules; each centrosome comprises two centrioles, which are composed of nine triplets of microtubules. The two centrioles are arranged perpendicularly and surrounded by an amorphous mass of dense material (the pericentriolar material) [30]. As in microtubules, an electric field would be generated by synchronized oscillation between the α and β tubulins within the microtubule triplet of the centrioles (Figure 2). 

### Electric fields in centrosome separation and bipolar spindle body assembly

Mechanisms of centrosome separation and bipolar spindle body assembly have been discussed in a recent review [31]. The process is still incompletely understood. Plus end-directed motor proteins such as kinesin 5 and minus end-directed motor proteins such as dynein are known to play dominant roles in centrosome separation and spindle assembly. However, centrosomal microtubules and microtubules of the nuclear envelope (NE) and cellular cortex need to move into close proximity for motor proteins to attach to both so they can generate the pulling forces. The current models assume a randomized mode of microtubule interaction, which is quite inefficient. For example, at a certain point a centrosome would have to stop moving until certain microtubules had grown sufficiently for appropriate bridging by motor proteins, particularly during prophase, when the centrosomes do not have many associated microtubules. When the electric fields of microtubules and centrosomes are considered, these structures are mutually attractive. Thus, centrosome movement along the microtubule networks of the cellular cortex and NE is more efficient. We can also envision a more autonomous mode of microtubule lattice formation within the cellular cortex and NE.

### Electrical properties of duplicated chromosomes

Andrews et al. have studied the effects of high frequency (range 2 to 50 MHz) electric fields on mammalian (human and Chinese hamster) chromosomes in vitro. They showed that such chromosomes can be oriented, aligned and translated by an oscillating electrical force. They also observed that above certain threshold field strengths the chromosomes orient themselves with their long axes along the field direction. The dependence of this threshold on frequency was measured and was found to be much larger at low than at high frequencies [32]. Using electric dichroism experiments, Crothers reported permanent dipole moments in dinucleosomes linked by 140 and 175 base pairs of DNA [33]. Jian Sun et al. suggested an electrostatic mechanism of nucleosomal array folding, revealed by computer simulation, which explains the salt-dependent chromatin fiber conformations [34]. Schalch et al. reported that the X-ray structure of an oligonucleosome revealed that linker DNA elements zigzag back and forth between two stacks of nucleosome cores, forming a truncated two-start helix, and do not follow a path compatible with a one-start solenoidal helix [35]. Grigoryev et al. reported evidence for heteromorphic chromatin fibers, showing that the 2-start zigzag topology and the type of linker DNA bending that defines solenoid models may be simultaneously present in a structurally heteromorphic chromatin fiber with a uniform 30 nm diameter [36].

However, the physical mechanisms that regulate higher order packaging of M phase chromosomes are still not well characterized. Here we present a hypothesis of chromosome compaction. We apply a pulse-coupled oscillation clustering model to the dynamic events of chromosome packaging and inter-/intra-chromosomal organization. During chromosomal packaging, differentially compacted regions form partially synchronized electric oscillators interacting with an elastic electromagnetic field. According to the physical pulse-coupled oscillator model, unsynchronized pulse-coupled oscillators with proximal natural frequencies form synchronized oscillation clusters at a given coupling strength. As the coupling strength increases, these synchronized oscillation clusters merge with each other [37-40].

**Figure 3:**
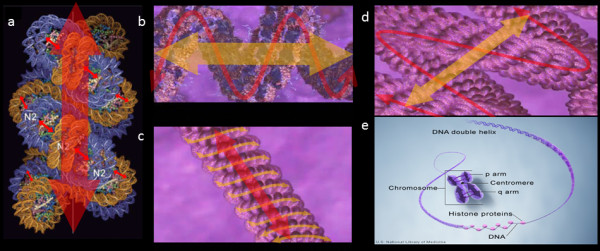
**(a) The small red arrows indicate the electric oscillations generated between the neighboring histone octamers by excitation of entropic energy within the cell nucleus.** The big red arrow represents the electric field generated by the electric oscillation along the 30 nm chromatin fiber. (**b**-**d**) Schematic illustration of several orders of oscillation coupling and clustering of EMFs in chromatin fibers, which facilitate the multi-step event of M phase chromosome packaging. The red and orange arrows indicate the multiple orders of EMFs generated during chromosome packaging. (**e**) The purple arrows indicate the EMFs of compacted M phase chromosome arms; the purple cycles indicate coupling of EMFs. The duplicated chromosome arms hold a juxtaposed position.

During M phase chromosome compaction, the 30 nm chromatin fiber is initially formed by the electrostatic forces between neighboring nucleosomes. Under intracellular stochastic energy excitation, electric dipolar oscillation would be generated between neighboring nucleosomes. After oscillation synchronization and coupling, regulated electric oscillation is generated along the 30 nm chromatin fiber, and the oscillation coupling process further compacts that fiber [12-14,41]. This facilitates further packing into the 300 nm fiber; the electric field bends according to the physical curvature of the compacting 30 nm fiber, generating an oscillating electromagnetic field that goes through the 300 nm chromatin fiber. After the second round of oscillation synchronization and coupling, the 300 nm fiber becomes compacted and coiled into the 250 nm chromatin fiber, along which the third order of electromagnetic field is generated; this round of oscillation coupling and clustering facilitates the packing of the 250 nm chromatin fiber into the 700 nm chromosome arms. The coiling electromagnetic field of the 250 nm chromatin fiber generates the electromagnetic field of a chromosome arm [42,43] (Figure 3). We speculate that the source of the dipolar electric oscillation between neighboring nucleosomes is the variety of intracellular entropic forces, and the direction of oscillation primarily depends on the zigzag arrangement of neighboring nucleosomes along the 30 nm chromatin fiber. Each of the M phase chromosomal arms can be viewed as a partially synchronized oscillation cluster. Under intracellular energy excitation, partially synchronized electric fields can be generated for each chromosomal arm, and these orient the dynamics of the chromosome when they interact with the electric field generated by the spindle microtubules. 

Theoretically, the oscillation clustering model explains the closely juxtaposed configuration of duplicated chromosomes during M phase, which is counter-intuitive from the perspective of electrostatic repulsion between duplicated chromosome arms. As the homologous chromosomal regions develop synchronized oscillation clusters with identical natural frequencies, they tend to cluster together. The same scenario could apply to synapsis during meiosis; the electric oscillations of homologous chromosomes couple with each other, preventing synapsis between non-homologous chromosomes.

### Electric interactions during mitosis and meiosis

**Figure 4:**
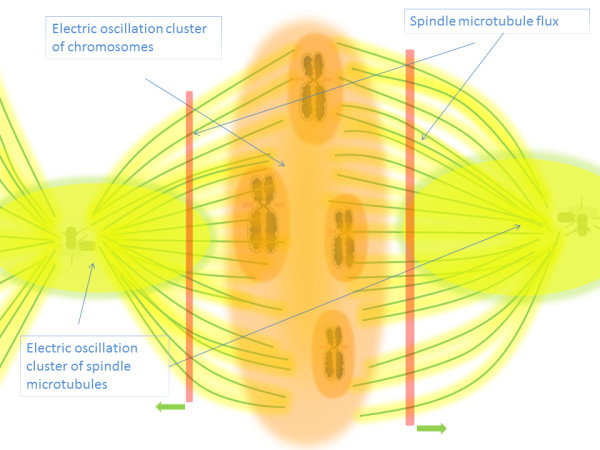
**Schematic illustration of the electric interactions between spindle body microtubules and chromosomes facilitating congression through oscillation clustering, and spindle body pole-ward flux during mitosis; the green arrows indicate the direction of the pole-ward flux** .

The intracellular electric fields described in the foregoing sections could facilitate several cellular events during mitosis and meiosis. First, the metaphase bipolar spindle microtubules are formed through microtubule nucleation from the γ tubulin ring complexes (γ-TuRCs) at the centrosomes and retrograde delivery of peripheral microtubules by motor proteins [31,44-46]. The electric fields of microtubules and centrosomes could facilitate spindle microtubule assembly through electric interactions. Secondly, the unidentified polar-wind or ejection force of the spindle body is likely to be generated by interactions between the electric fields of the spindle body and chromosomes [1-3]. In this case, the bipolar spindle body and chromosomes can be viewed as two oscillating clusters with different average oscillating frequencies. Given the oscillation clustering model, a partially entrained system of oscillators with similar frequencies preferentially cluster with each other at a given coupling strength [37-40]. So the clustering of electric fields of spindle microtubules would result in the repulsion of the electric field of the chromosomes, which pushes the chromosomes from the proximal regions of centrosomes out of the spindle body. Congression at the central plate regions of a dividing cell can also be viewed as oscillation clustering of the electric fields of chromosomes. The processes may also be viewed as a dynamic electric phase, which indicates that the intensity of the electric field changes in different subcellular regions during metaphase. Cell organelles with different electric field intensities would automatically locate themselves according to the electric phase. In addition, the pole-ward flux of spindle microtubules during metaphase could be driven by the electric locking of those microtubules within the spindle body, which means that the synchronized electric fields of the spindle body would hold the physical position of a spindle microtubule growing at the plus end and depolymerizing at the minus end at the same time within a dividing cell [5] (Figure 4). 

According to the physical organization of the duplicated chromosome arms, the condensed electric chromosomal fields around the centromeric regions could attract microtubule fragments to the sister kinetochores through electric interaction, which is consistent with observations of kinetochore movement along uncaptured microtubules, forming K-fibers (kinetochore associated microtubules) [47]. Thus, electric interactions between chromosomes and microtubules may also facilitate K-fiber capture by kinetochores. The chromosome oscillation observed during congression could be explained as the turbulence of chromosome arms passing through the chaotic electric landscape of two astral microtubule networks.

In meiosis, the kinetochores are positioned at one side of the duplicated chromosome dimers, so the two sister chromosomes do not separate. The electric oscillation clustering between homologous chromosomal regions results in synapsis and recombination between homologous chromosomes; the electric fields generated by two duplicated homologous chromosomes can be viewed as two identical partially-entrained electric oscillation clusters, constituted by sub-chromosomal clusters throughout the chromosome arms. Such clusters in homologous chromosomes share identical electric frequencies, so the close juxtaposition between homologous chromosomes at synapsis is achieved through electric clustering and coupling among them. Synapsis does not occur during mitosis probably because the chromosome configuration caused by the opposing outward-pulling forces of the kinetochores at the opposite sides of duplicated centromere disfavors inter-chromosomal electric attraction. In addition, this event may be regulated by synaptonemal complex proteins [48].

Magidson et al. reported that chromosomes adopt a toroidal/ring shape organization of after NE breakdown, which facilitates spindle assembly during M phase [47]. Their observation matches the electric model at several points: the ring shape organization could be generated by the electric interaction between M phase chromosomes and the spindle body, and the interplay between the electric fields of the chromosome ring and spindle body microtubules promotes the capture of microtubules by kinetochores.

## Discussion

Numerous reports indicate that extremely low frequency electric fields can increase the risks of certain types of cancer [49]. Micronuclei (MN) in buccal mucosal cells, comprising acentric fragments or complete chromosomes that fail to attach to the mitotic spindle during cytokinesis, are increased in people chronically exposed to extremely low frequency electric fields [50]. Research by Hardell et al. indicates increased brain tumor risks with latency time and cumulative mobile or cordless phone use [51]. Volkow et al. reported that 50-minute cell phone exposure was associated with increased brain glucose metabolism in the region closest to the antenna [52]. However, the exact cellular biophysical pathways that relay very low frequency electric radiations to genetic alterations that lead to cancer are not well characterized. From our theoretical point of view, chronic exposure to extremely low frequency electric fields would intervene in several key steps of mitosis and neuronal cell physiology, potentially resulting in an increased risk for cancer.

To characterize these intracellular electric fields and study their cellular functions further, biophysicists should develop more detailed mathematical and physical models for chromosome electric fields and their role in M phase chromosome compaction, to allow these fields to be described and calculated more precisely and to predict the dynamics of related cellular events. The dynamic of changes of the electric fields in a living cell during mitosis and other cellular processes could be visualized using live cell imaging technologies such as nano-sized voltmeters [53]. It would be particularly interesting to observe microtubule self-organization under energy excitation in vitro, which would allow us to observe the dynamics of microtubule movements directly through electric interactions. These insights will help us to understand the molecular mechanisms of signal pathways better and to elucidate cellular super-macromolecular behavior, cell organelle organization and functions, intra- and inter-cellular communications, tissue morphogenesis, embryo development, neurobiology, and oncogenesis, and finally to advance our knowledge about life to a new level.

## Competing interests

The authors declare they have no competing interests.

## Authors’ contributions

Yue Zhao conceived the general concepts and wrote the article; Qimin Zhan shared insights and gave advice. Both authors read and approved the final version of the article.
